# Sulphonamide and Trimethoprim Resistance Genes Persist in Sediments at Baltic Sea Aquaculture Farms but Are Not Detected in the Surrounding Environment

**DOI:** 10.1371/journal.pone.0092702

**Published:** 2014-03-20

**Authors:** Windi Indra Muziasari, Satoshi Managaki, Katariina Pärnänen, Antti Karkman, Christina Lyra, Manu Tamminen, Satoru Suzuki, Marko Virta

**Affiliations:** 1 Department of Food and Environmental Sciences, University of Helsinki, Helsinki, Finland; 2 Department of Environmental Sciences, Musashino University, Tokyo, Japan; 3 Centre for Marine Environmental Studies (CMES), Ehime University, Matsuyama, Ehime, Japan; Institut National de la Recherche Agronomique, France

## Abstract

Persistence and dispersal of antibiotic resistance genes (ARGs) are important factors for assessing ARG risk in aquaculture environments. Here, we quantitatively detected ARGs for sulphonamides (*sul1* and *sul2*) and trimethoprim (*dfrA1*) and an integrase gene for a class 1 integron (*intI1*) at aquaculture facilities in the northern Baltic Sea, Finland. The ARGs persisted in sediments below fish farms at very low antibiotic concentrations during the 6-year observation period from 2006 to 2012. Although the ARGs persisted in the farm sediments, they were less prevalent in the surrounding sediments. The copy numbers between the *sul1* and *intI1* genes were significantly correlated suggesting that class 1 integrons may play a role in the prevalence of *sul1* in the farm sediments through horizontal gene transfer. In conclusion, the presence of ARGs may limit the effectiveness of antibiotics in treating fish illnesses, thereby causing a potential risk to the aquaculture industry. However, the restricted presence of ARGs at the farms is unlikely to cause serious effects in the northern Baltic Sea sediment environments around the farms.

## Introduction

Aquaculture production is increasing worldwide as a source of fish for human consumption. Aquaculture introduces land-derived microbes, nutrients, metals and other chemicals such as antibiotics to the water environment. The prophylactic and therapeutic use of antibiotics results in the occurrence of antibiotic-resistant bacteria and antibiotic resistance genes (ARGs) in the aquaculture environment [1], [2]. This may lead to seawater and the sediment becoming reservoirs for ARGs [3]. The ARGs in aquaculture environments can be transferred horizontally among microbes and ultimately be transferred to fish pathogens [4]. Thus, the presence of ARGs in aquaculture environments may lead to inefficiency in treating fish diseases using antibiotics [5]. To avoid production losses in the fish-farming industry, it is important to control the occurrence and spread of ARGs in aquaculture facilities.

The spreading of ARGs in the environment is mediated by horizontal gene transfer (HGT) [6]. Therefore, genes associated with HGT should also be examined when determining the prevalence of ARGs in the environment. Integrons can contribute to the occurrence of HGT of ARGs in bacterial populations [7], [8] as a consequence of possessing a site-specific recombination system capable of capturing gene cassettes containing ARGs [9]. Integrons can be carried by mobile genetic elements such as transposons and plasmids that promote their wide distribution within bacterial communities [8]. Class 1 integrons, which contain an *intI1* gene encoding an integrase of the tyrosine recombinase family, are known to carry gene cassettes containing ARGs [10]. Class 1 integrons have been found in cultured fish pathogens [11] and from cultured bacteria in the aquaculture environments [4], [12].

Sulphonamides potentiated with trimethoprim or ormethoprim and florfenicol are some of the antibiotics commonly used in aquaculture [5]. Consequently, the presence of several antibiotic-resistant bacteria in aquaculture environments has been reported previously using culture-dependent methods. Bacteria of the *Actinobacter* [13] and *Bacillus* genera [14] have been observed to carry the resistance genes to sulphonamides and *Aeromonas* and *Pseudomonas* to florfenicol [15]. Also aquatic bacteria of the genera *Proteus* and *Pseudomonas* can carry the resistance genes to both sulphonamides and trimethoprim [4]. However, culture-dependent methods may introduce bias when determining the prevalence of ARGs due to the inability to cultivate the majority of bacteria from environmental samples [16].

Culture-independent methods, including the measurement of gene copy numbers by quantitative polymerase chain reaction (qPCR), give a less biased estimation of the ARG amounts in the environment. qPCR has been widely used to study ARGs in environmental samples [17]–[19] and aquaculture environments [14], [20]. However, little is known about the long-term persistence of ARGs at aquaculture sites and their dispersal to the surrounding environment [20]. To investigate these aspects, we collected sediment samples below two open-cage fish farms located in the Turku Archipelago, Finland in the northern Baltic Sea during the summers of 2006 to 2012. Sediment samples were also collected 200-m and 1000-m from fish farms, as well as from transect sites at 200-m intervals up to 1000 m from one farm to observe the dispersal of ARGs to the surrounding sediment environment. We quantified ARGs for sulphonamides, trimethoprim, and florfenicol and an integrase gene of class 1 integrons using qPCR. The antibiotic concentrations (sulphamethoxazole, sulphadiazine and trimethoprim) were measured using liquid chromatography-mass spectrometry (LC-MS). Our findings show that the ARGs were abundant and persistent in the sediments below the fish farm cages during the 6-year observation period, but were not detected in the sediments even at the closest 200-m distance from the cages. Moreover, a correlation was found between the amount of class 1 integrons and the *sul1* gene.

## Results

### Antibiotic resistance genes and class 1 integrons in sediments

**Figure 1:**
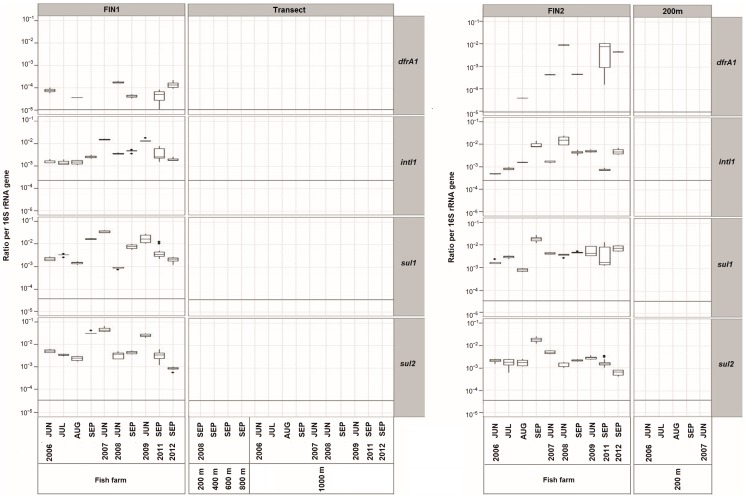
Gene copy numbers in the sediments. The ratios of the trimethoprim resistance gene (*dfrA1),* sulphonamide resistance gene *(sul1* and *sul2)* and an integrase gene of a class 1 integron (*intI1)* to the 16S rRNA gene copies were elevated in the farm sediments. None of these genes were detected in sediment samples at the closest 200-m distance from the two farms during the 6-year observation period. The missing data values in the plot mean that the respective gene copy numbers were below the limit of detection in the qPCR assays. The gene quantification limit normalized to the average numbers of the 16S rRNA gene copies is indicated by a grey line.

The detection of trimethoprim resistance genes (*dfrA1, dfrA2, dfrA5, dfrA12, dfrA15, dfrA16, dfrA17,* and *dfrA19),* sulphonamide resistance genes (*sul1, sul2* and *sul3*), florfenicol resistance gene (*floR*) and an integrase gene (*intl1*) of class 1 integrons was done for six sediment samples chosen from northern Baltic Sea fish farm sites, using standard PCR. From the targeted ARGs, the *dfrA1, sul1, sul2* and *intI1* genes were found at two fish farms (FIN1 and FIN2). The amounts of the genes detected were measured, using qPCR in all 51 sediment samples. The copy numbers of the four genes were under the detection limit in every sediment sample taken 200 m to 1000 m from the farms as shown in Figure 1. The dispersal of the ARGs was not considerable, since the genes were not detected even at the closest 200-m distance from each farm.

The *sul1, sul2* and *intI1* genes were present in every sample and the *dfrA1* gene in most samples from the FIN1 and FIN2 farms throughout the 10 sampling times from 2006 to 2012 (Figure 1). The *sul1*, *sul2* and *intI1* gene copy numbers at the two farms were similar, with about 10^−3^–10^−2^ copies in proportion to the 16S ribosomal RNA (rRNA) gene copies. The *dfrA1* gene copy numbers varied approximately 10^−5^–10^−2^ copies in proportion to the 16S rRNA gene copies. The abundance of the *dfrA1* gene was lower than and significantly different from that of the *sul1* (*P*<0.001), *sul2* (*P*<0.001) and *intI1* (*P*<0.001) genes.

### Correlation between ARGs and class 1 integrons in the sediments

**Figure 2:**
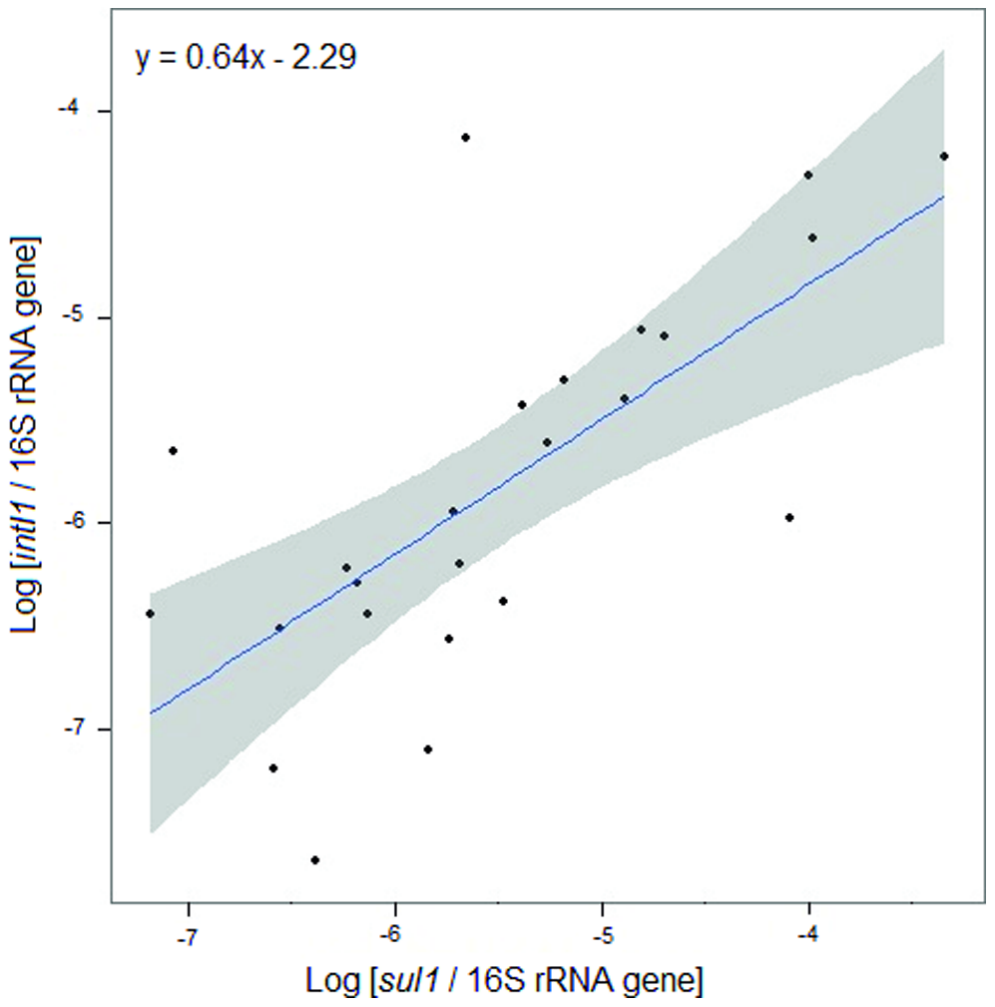
Correlation analysis. Linear regression model with log-transformed variables between the *intI1* and *sul1* gene copy numbers in the sediments below the northern Baltic Sea farms (*F_1,22_* = 19.39; *P* = 0.000225; *R^2^* = 0.47). Each point represents the average ratio of a gene copy number normalized to the 16S rRNA gene copy number in every sediment sample. The blue line indicates the regression model and the grey area the 95% confidence intervals.

Linear regression analysis was performed to test whether the copy number of the class 1 integron was correlated with any of the three ARGs detected (*dfrA1*, *sul1* and *sul2*) and thus could have played a role in the prevalence of the ARGs. Significant correlation (*F*_1,22_  = 19.39; *P* = 0.000225; *R^2^* = 0.47) was found only between the average copy numbers of the *intI1* and *sul1* genes (Figure 2). The prevalence of the *sul1* gene in the Baltic Sea farm sediment may therefore be associated with class 1 integrons.

### Antibiotic concentrations in the sediments

The sulphamethoxazole, sulphadiazine, and trimethoprim concentrations were measured, using LC-MS analysis to estimate the presence of selection pressure in the sediments. The antibiotic concentrations in the sediments are shown in Table 1. All the antibiotic concentrations measured in the farm sediments were very low (1.5–101 ng g^−1^ of dry sediment) and some even below the detection limit (<1 ng g^−1^ of dry sediment). The antibiotic concentrations were also below the detection limit in the sediments taken 200 m to 1000 m from the farms. Hence, there was no clear selection pressure in the sediments.

**Table 1:** Antibiotic concentrations in sediments.

Sites	Sampling times	Sulfamethoxazole (ng g^−1^)	Sulfadiazine (ng g^−1^)	Trimethoprim (ng g^−1^)
FIN1-Farm	JUN 2007	<1	<1	<1
	JUN 2009	3.6	2.3	<1
	SEP 2011	7.1	3.2	1.5
1000-m distance from FIN1 farm	JUN 2009	<1	<1	<1
	SEP 2011	<1	<1	<1
FIN2-Farm	JUN 2007	41	23	5.7
	SEP 2011	101	47	8.9
200-m distance from FIN2 farm	JUN 2007	<1	<1	<1

The detection limit  =  1 ng g^−1^ of dry sediment. The relative standard deviations of the target antibiotics ranged between 8% and 13%.

## Discussion

Our results showed that the sulphonamide resistance genes (*sul1* and *sul2*) and trimethoprim resistance gene (*dfrA1*) were persistent in the Baltic Sea farm sediments during the 6-year observation period. We assume that the Baltic Sea farms have relatively small impact from human habitats and agriculture and therefore municipal and agricultural ARG sources can be excluded. The antibiotics and organic matter from uneaten fish feeds and fish excrement can enter the sediments directly from the water since there is no purification process in the open-cage farming system. Thus, the selection for the resistant bacteria and ARGs may have occurred in the medicated fish feeds [20] or inside the fish intestines and fish faeces that entered the sediments [21], [22], or selection through the antibiotics present in the sediments [3], [22]. However, the LC-MS results indicated low concentrations of sulphonamides and trimethoprim, suggesting that there was no clear selection pressure in the farm sediments. Furthermore, sulphonamides are decomposed by chemical and biological factors with half-lives of 7–85 days [23]. The persistence of ARGs in the farm sediments may, therefore, have been due to a constant introduction of ARGs from external sources, such as uneaten medicated fish feeds and fish faeces [16].

On the other hand, very low concentrations of the antibiotics may also play a role in maintaining the ARGs in the farm sediments by selection and enrichment of resistant bacteria by a subinhibitory antibiotic concentration, which was shown in a previous study [24]. Moreover, the presence of antibiotics in a subinhibitory concentration may induce the HGT system in bacterial communities [25], which further increases the prevalence of ARGs. Therefore, the potential for low antibiotic concentrations in maintaining resistant bacteria also needs to be examined to further understand the persistence of ARGs in northern Baltic Sea farm sediments.

In this study, the dispersal of ARGs (*sul1, sul2 and dfrA1*) from the Baltic Sea aquaculture farms to the surrounding sediments was not detected. Previous work from the same fish farms locations shows similar results; the four tetracycline resistance genes studied were not detected in the sediments even at the closest 200-m distance from the farm cages [20]. These results suggest that the resistance genes are potentially a problem for the fish-farming industry, but impact less the surrounding sediment environment in the northern Baltic Sea.

The copy number of the class 1 integron *intl1* was significantly correlated with the *sul1* gene copies in the farm sediments. This was expected, since the *sul1* gene is one of the backbone genes of the 3′-conserved segments in class 1 integrons [10]. Class 1 integrons are, therefore, likely involved in the prevalence of the *sul1* gene in Baltic Sea fish farm sediments. Similar correlations between the copy numbers of the *intI1* and *sul1* genes have been observed in riverine sediments in Haihe, China [18] and in Colorado, USA [19]. Only *dfrA1* of the eight trimethoprim resistance genes analysed (*dfrA1, dfrA2, dfrA5, dfrA12, dfrA15, dfrA16, dfrA17 and dfrA19*), which are commonly associated with class 1 integrons [6], was detected in the Baltic Sea farm sediments. There was no significant correlation between the amounts of the *intI1* gene of class 1 integrons and the *dfrA1* gene copies, suggesting that the *dfrA1* gene was not associated with class 1 integrons in the farm sediments. The prevalence of the *dfrA1* gene may have been mediated by other mobile elements or even independently of them [9].

The copy number of *dfrA1* was significantly lower than that of *sul.* Although the amount of *dfrA1* genes was lower than the amount of *sul* genes, the prevalence of both genes in the aquaculture environment deserves equal focus, because sulphonamides and trimethoprim are used in combination as so-called potentiated sulphonamides [5], [26]. While many studies have demonstrated sulphonamide resistance in aquaculture environments [17], to our knowledge, this is the first study reporting qPCR measurements of the trimethoprim resistance gene (*dfrA1*) in aquaculture-impacted sediment samples.

In conclusion, the persistence of ARGs in farm sediments may lead to problems in the efficiency of antibiotics used to treat fish diseases and eventually to production losses at the fish farms. It is important for the fish-farming management to control the use of antibiotics to avoid the emergence of ARGs at the farms. Since the ARGs were not detected 200 m and 1000 m from the farms, their presence at the farms is unlikely to cause serious effects in the aquatic environment surrounding fish farms in the northern Baltic Sea in the current environmental conditions. However, a change in environmental conditions or an extended exposure to nearby fish farming activity could conceivably lead to an emergence of antibiotic resistance genes in the future. The sources and the spread of ARGs in aquaculture process chains should also be studied, as well as their potential risk to human health.

## Materials and Methods

### Study site and sampling

The sediment samples were collected from two fish farms (FIN1 and FIN2) and nearby areas located in the Turku Archipelago, Finland in the northern Baltic Sea from 2006 to 2012. The northern Baltic Sea is a unique brackish water marine environment (mean salinity: 6.7 parts per thousand) and no tide [27]. The sampling locations are described in Table 2. Both the FIN1 and FIN2 farms use open-cage systems in which the fish are kept in net cages that allow free transfer of uneaten fish feeds and fish excrement from the cages to the surrounding waters and eventually to the sediments. The farms raise European whitefish (*Coregonus lavaretus* (L.)) and rainbow trout (*Oncorhynchus mykiss* (Walbaum)). Each farm produces approximately 50 tons of fish annually. The record amount of antibiotics used at the fish farms was not available.

**Table 2:** Sampling sites and their descriptions.

Sites	Mean value at sampling times^a^			Locations
	depth (m)	T (°C)	pH	
FIN1Farm	6 (+/−SD 1)	15.3 (+/−SD 3.2)	7.6 (+/−SD 0.5)	Located in the middle of a 400-m-wide strait
Distance from FIN1 farm	8 (+/−SD 0.4)	15.1 (+/−SD 2.7)	4.2 (+/−SD 1.6)	A site 1000-m distance from the FIN1 farm. In addition, a transect was sampled along the strait of the FIN1 farm at 200-m intervals up to 1000 m
FIN2-Farm	7.4 (+/SD 1.9)	16 (+/−SD 2.7)	7.9 (+/−SD 0.5)	Located next to the seashore in an 800-m-wide strait
Distance from FIN2 farm	5.1 (+/SD 2.6)	16.5 (+/−SD 2)	8.1 (+/−SD 0.3)	A site 200-m distance from the FIN2 farm

a Mean values of depth, temperature (T) and pH were measured from bottom seawater at sampling sites located in the archipelago area in the northern Baltic Sea.

Northern Baltic Sea aquaculture farms operate only in summer. Sampling was done 10 times during the 6-year observation: June, July, August and September 2006, June 2007, June and September 2008, June 2009, September 2011 and September 2012. In addition, transect interval samples were collected at sites 200-m up to 1000-m distance from the FIN1 farm on September 2008. Three replicate samples were collected in each year from 2006 to 2009 and the replicates from each year were pooled. In 2011 and 2012, three biological replicates were individually collected. In all, 51 samples from the FIN1 farm and FIN2 farm and surrounding areas were collected using a Limnos sediment probe (Limnos Ltd., Turku, Finland). Each sample was homogenized manually inside a zipper storage plastic bag and immediately frozen on dry ice. The sediments were stored at −80°C until DNA extraction.

### DNA extraction

The environmental total DNA was extracted from 0.5 g wet weight sediment, using the FastDNA® SPIN kit for soil (MP Biomedicals, Illkrich, France). The standard protocol was modified by adding an extra washing step with 5.5 M of guanidine thiocyanate (Sigma Life Science, Steinheim, Germany), according to the manufacturer's instructions for removing humic acids. The DNA quality and concentration were analysed with a Nanodrop 1000 spectrophotometer (Thermo Scientific, Wilmington, DE, USA). The extracted DNA was stored at −20°C.

### Standard PCR

The PCR primers and conditions for detecting the presence of targeted genes in the sediments were described previously: sulphonamide resistance genes (*sul1, sul2* and *sul3*) [17], trimethoprim resistance genes associated with mobile elements (*dhfr1, dfrII, dfrV, dhfrIX, dfrXII, dhfrXV, dfr16, dfr17* and *dfrA19*) [28], currently known as *dfrA1, dfrA2, dfrA5, dfrA9, dfrA12, dfrA15, dfrA16, dfrA17* and *dfrA19* [10], florfenicol resistance gene (*floR*) [28] as well as the integrase gene of the class 1 integron (*intI1*) [29]. Six sediment samples from the FIN1 farm, FIN2 farm and 1000-m distance from the FIN1 farm, taken during years 2007 and 2011, were chosen for gene detection in PCR.

The 25-μl PCR reactions consisted of 1× Taq buffer with (NH_4_)2SO_4_, 2 mM MgCl_2_, 0.2 mM of each deoxyribonucleotide triphosphate (dNTP), 50 U Taq DNA polymerase, recombinant (Finnzymes, ThermoFisher Scientific, Espoo, Finland), 0.2 μM of each primer (Oligomer Oy, Helsinki, Finland) and the DNA template. The negative controls had nuclease-free water and were done for every PCR reaction. All the PCR reactions were done in triplicate using a PTC-200 thermal cycler (MJ Research, Watertown, MA, USA). The PCR products were purified, using QIAquick PCR purification kit (Qiagen, Hilden, Germany) and sequenced by the DNA sequencing service at the Institute of Biotechnology (University of Helsinki, Finland) to confirm the sequences of the PCR product.

### Quantitative PCR measurement

The primers for the *dfrA1* gene [28] and the *intI1* gene [29] used in standard PCR were not optimal for the qPCR assays in this study. Thus, new primer sets were designed. The *dfrA1* and *intI1* gene sequences (accession numbers indicated in Table 3) were submitted to Primer3 v.2.3.4 to produce primer sets with melting temperatures above 60°C and amplifying 150–250 base-pair (bp)-long fragments. The primers designed were compared with known gene sequences that were retrieved from GenBank and aligned with mafft [30] to choose primer sets that are located in conserved regions. Basic Local Alignment Search Tool (BLAST) analysis of the primer sequences against the National Center for Biotechnology Information (NCBI) database was performed to avoid nonspecific amplification.

**Table 3:** Primers used for qPCR assays.

Target gene	Primers	Sequence 5′ – 3′	Ta (°C)	Product (bp)	References
*sul1*	sulI-FW	CGC ACC GGA AAC ATC GCT GCA C	64	163	[13]
	sulI-RV	TGA AGT TCC GCC GCA AGG CTC G			
*sul2*	sulII-FW	TCC GAT GGA GGC CGG TAT CTG G	60	191	[13]
	sulII-RV	CGG GAA TGC CAT CTG CCT TGA G			
*sul3*	sulIII-FW	TCC GTT CAG CGA ATT GGT GCA G	60	128	[13]
	sulIII-RV	TTC GTT CAC GCT TTA CAC CAG C			
*dfrA1*	dfrA-q-FW	TTC AGG TGG TGG GGA GAT ATA C	60	150	This Study
	dfrA-q-RV	TTA GAG GCG AAG TCT TGG GTA A			[NC_006385.1]
*floR*	floR-right	TCG TCA TCT ACG GCC TTT TC	60	188	[28]
	floR-left	CTT GAC TTG ATC CAG AGG GC			
*intI1*	intI1-a-FW	CGA AGT CGA GGC ATT TCT GTC	60	217	This study
	intI1-a-RV	GCC TTC CAG AAA ACC GAG GA			[NC_017659.1]
16S rRNA	pA	AGA GTT TGA TCC TGG CTC AG	60	350	[34]
	358R	CTG CTG CCT CCC GTA GG			[35]

Plasmids R388, RSF1010 and pUV441 were used as the *sul1, sul2,* and *sul3* gene standards. Presynthesized pUC57 vectors containing either the entire 459-bp sequence of the *dfrA1* gene or a 250-bp fragment of the *floR* gene were ordered from GenScript (Piscataway, NJ, USA). The chromosomal DNA of *Escherichia coli* K12 was used as the 16S rRNA gene standard. The plasmid standards were linearized and purified. The standard copy numbers per μl were calculated using the estimated molar mass for the DNA bp (650 Da/1 bp). Determination of the qPCR efficiency was done, using a five-point 10-fold dilution series. In addition, inhibition tests were performed as previously described [31] to observe whether the sediment samples had the same amplification efficiency as the standard. The average inhibition was 2.5% (+/− SD 1.9%).

The qPCR was performed using a 7300 real-time PCR system (Applied Biosystems, Foster City, CA, USA). The 20-μl qPCR reactions contained 1× DyNAmo Flash SYBR Green Master Mix (Thermo Scientific) with 1× of ROX passive reference dye, 0.625-μM primers for the targeted gene listed in Table 3 and freshly diluted DNA sample. Nuclease-free water was added instead of DNA samples for the no template control (NTC) sample. The qPCR programs consisted of 7 min at 95°C, 40 cycles of 10 s at 95°C, 30 s at the annealing temperatures (Ta) listed in Table 3 and melting curve analysis. Three technical replicates of each sample, NTC and standard dilution were performed in each measurement and the measurements were repeated once to determine the reproducibility of the qPCR assays. Based on previous studies of the validation of qPCR assays [32], [33], each assay was performed until the qPCR assay characteristics were achieved as described in Text S1. The limit of quantification (LOQ) of the qPCR assay was 5.92×10^−5^ copies in proportion to the average number of 16S rRNA gene copies for *sul1*, *sul2*, *sul3* and *floR*, 1.18×10^−5^ copies in proportion to the average of 16S rRNA gene copies for *dfrA1* and 2.96×10^−4^ copies in proportion to the average number of 16S rRNA gene copies for *intI1*. Data analysis was performed manually, using the 7300 System SDS v.1.2 Software (Applied Biosystems).

### Statistical analysis

Student's t-test was performed to determine whether the copy numbers of the ARGs from all farm samples varied significantly. The correlation between the average gene copy numbers of the integrase gene of the class 1 integron and the ARGs detected in the farm sediments was analysed, using linear regression. The t-test and the linear regression with log-transformed variables were performed using RStudio v.0.97.168 (RStudio, Boston, MA, 2012). All models were considered to be significant at p-values less than 0.05.

### Analytical methods for antibiotic quantification

The liquid chromatography analyses were performed with a Hewlett-Packard 1100 (Hewlett-Packard Co., Palo Alto, CA, USA). The reagents used and extraction methods are described in Text S2. The antibiotics were separated on a reverse-phase column (YMC Pro C18, 3 μm, 150 mm×2 mm; YMC America Inc., Allentown, PA, USA) operated at 30°C at a flow rate of 0.15 ml min^−1^. The mobile-phase solvents were water-acidified with 1% (v/v) formic acid (eluent A) and methanol-acidified with 1% (v/v) formic acid (eluent B) to a pH of 2.5. The HPLC gradient programmes are contained in Table S1 in Text S2. The antibiotics were detected with a Micromass Quattro Ultima triple-quadrupole MS (Micromass, Milford, MA, USA), equipped with electrospray ionization. The analyses were performed in the positive ion mode. The protonated molecular ion ([M+H]+) of the compounds was selected as the parent ion. Detection was performed in the multiple reaction-monitoring mode, using the two most intense and specific fragment ions. Table S2 in Text S2 lists the optimized conditions of the individual analytes. To identify the antibiotics, we compared the retention times and the area ratios of the two product ions in each sample with the average retention time and peak ratios of the standards in all measurements. The criteria difference between the samples and the standard was within 0.3 min for the retention time and 20% for the area ratio of the two product ions. The concentrations of the samples were calculated by an external standard method, based on the peak area of the sum of the two product ions monitored. The calibration lines of five concentration points (10, 20, 30, 40, and 50 μg l^−1^ in water-methanol [1∶1]) of the individual antibiotics were used for quantification. The linearity of the calibration curve in this range was confirmed (*R* ^2^>0.99). The final concentrations of most of the samples in the vials were within the range of the calibration lines.The LOQs were defined as 10 times the noise level of the baseline in the chromatograms signal to noise (S/N) ratio and were 1 ng g^−1^.

### Ethics statement

No specific permits were required for the field study described. The sampling did not affect any protected or endangered organisms.

## Supporting Information

Text S1 **Quantitative PCR (qPCR) measurement.**
(DOCX)

Text S2 **Analytical methods for antibiotic quantification.**
(DOCX)
