# Supplementary material for: Sulphonamide and Trimethoprim Resistance Genes Persist in Sediments at Baltic Sea Aquaculture Farms but Are Not Detected in the Surrounding Environment
Source: PLoS One. 2014 Mar 20;9(3):e92702. doi: 10.1371/journal.pone.0092702 (PMC3961581; doi:10.1371/journal.pone.0092702)
Supplement: Text S1 — Quantitative PCR (qPCR) measurement. (DOCX) [file pone.0092702.s001.docx]

**Quantitative PCR (qPCR) measurement**

Each qPCR assay was performed until the following characteristics were achieved: PCR efficiency 90–110%, R^2^ > 0.99 and standard curve y-intercept of approximately 40. The qPCR assay of *sul1, sul2, sul3, dfrA1* and *floR* had NTC quantification cycle (Cq) values > 40 and the limit of detection (LOD) for the assays was 1 x 10^2^ genes per reaction at Cq values of approximately 33. For the *intI1* and 16S rRNA genes, the NTC had Cq values of approximately 33 and the LODs were 1.0 x 10^3^ genes per reaction at Cq values of approximately 30. Thus, the limit of quantification (LOQ) of the qPCR assay, which was calculated from the LOD and the dilution times of the DNA sample, was 5.92 x 10^-5^ copies in proportion to the average number of 16S rRNA gene copies for *sul1, sul2, sul3* and *floR*, 1.18 x 10^-5^ copies in proportion to the average number of 16S rRNA gene copies for *dfrA1* and 2.96 x 10^-4^ copies in proportion to the average number of 16S rRNA gene copies for *intI1*.

Data analysis was performed manually, using the 7300 System SDS v.1.2 Software (Applied Biosystems). Melting curve analysis was performed to confirm the specificity of the reactions. The qPCR reactions that did not yield specific peaks in the melting curve analysis were analysed, using electrophoresis on 1.5% (w/v) agarose gels. Samples that yielded nonspecific bands were discarded. The outlier Cq values from three technical replicates in every sample were removed and Cq standard deviations below 0.25 were used in the analysis to avoid results from technical measurement errors.
