# Supplementary material for: Sulphonamide and Trimethoprim Resistance Genes Persist in Sediments at Baltic Sea Aquaculture Farms but Are Not Detected in the Surrounding Environment
Source: PLoS One. 2014 Mar 20;9(3):e92702. doi: 10.1371/journal.pone.0092702 (PMC3961581; doi:10.1371/journal.pone.0092702)
Supplement: Text S2 — Analytical methods for antibiotic quantification. (DOCX) [file pone.0092702.s002.docx]

| **Analytical methods for antibiotic quantification**  **Reagents**  High-performance liquid chromatographic (HPLC)-grade methanol, water and ethyl acetate, analytical-grade sodium chloride, 28% ammonia solution, 47% sulphuric acid, 90% formic acid, sulphamethozaxole, sulphadiazine, trimethoprim and sulphamethozaxole-d^4^ were purchased from Wako Pure Chemical Industry, Ltd. (Tokyo, Japan).  **Sample preparation**  The sediment samples were analysed to quantify the amounts of sulphamethoxazole, sulphadiazine and trimethoprim according to methods previously described [1]. The samples were freeze-dried before extraction. Five hundred milligrams of freeze-dried sediment were extracted by accelerated solvent extraction (ASE) with a Dionex 200 instrument (Tokyo, Japan). The sediment was transferred to a stainless-steel extraction cell of 22-ml capacity and the void space was filled with diatomaceous earth, granular (Wako). The cells were heated to 100 °C for 5 min and the antibiotics extracted with a methanol-water mixture (50/50, v/v) at 100 bar. The flush volume was 40% of cell volume (viz., 8.8ml) over three static cycles. The extracts were collected in 50-ml amber vials. A surrogate standard was spiked directly on the sample in the extraction cell. The respective extracts were adjusted to pH 4 with sulphuric acid. Solid-phase extraction was performed in 6-ml Oasis HLB sorbent cartridges (200 mg; Waters Corp., Milford, MA, USA) at a flow rate of < 5 ml min^-1^. The cartridges were preconditioned with 2 × 1.5 ml of methanol-ethyl acetate (1:1), 2 × 1.5 ml of methanol containing 1% (v/v) ammonia and 2 × 1.5 ml of water adjusted to pH 4 with sulphuric acid. After extraction, the cartridges were washed with 5 ml of water-methanol (75:25) and dried in a nitrogen flow for 30 min. The analytes were then eluted with 2 × 1.5 ml of methanol-ethyl acetate (1:1) and 2 × 1.5 ml of methanol containing 1% (v/v) ammonia into a pear-shaped flask. The extracts were rotary evaporated to dryness at 40 °C under reduced pressure (∼100 mm Hg) and redissolved in 1 ml of water-methanol (1:1) and then separated by liquid chromatography/tandem mass spectrometry for the determination of antibiotics.  **Method validation**  The analytical precision was examined through four replicate analyses of 200-mg samples. The relative standard deviations of the target antibiotics ranged between 8% and 13%. For recovery studies, the recoveries of the spiked standards ranged between 71% and 90%. The LOQs were defined as 10 times the procedural blank value or 10 times the noise level of the baseline in the chromatograms if no peaks were detected in the procedural blank analysis.   1. Goebel C, Alma C, Howe C, Kazlauskas R, Trout G (2005) Methodologies for detection of hemoglobin-based oxygen carriers. J Chromatogr Sci 43: 39-46. | | | |
| --- | --- | --- | --- |
| **Table S1. The HPLC gradient programmes** | | | |
|  | | | |
| Gradient programmes: | Time (min) | A (%) | B (%) |
|  | 0 | 90 | 10 |
|  | 5 | 90 | 10 |
|  | 10 | 85 | 15 |
|  | 15 | 60 | 40 |
|  | 20 | 55 | 45 |
|  | 30 | 30 | 70 |
|  | 32.5 | 0 | 100 |
|  | 42.5 | 0 | 100 |
|  | 45 | 90 | 10 |
|  | 55 | 90 | 10 |
|  | 75 | 90 | 10 |

| **Table S2. Parent ions, product ions of the measured compounds** | | | |
| --- | --- | --- | --- |
|  | | | |
| Compound name | Parent ion (m z^-1^) | Product ions (m z^-1^) | |
| sulphamethozaxole | 254.2 | 156.1 | 107.9 |
| sulphadiazine | 251.2 | 156.0 | 107.8 |
| trimethoprim | 291.1 | 123.1 | 230.0 |
| sulphamethozaxole-d4 | 258.0 | 160.1 | 112.0 |
